# Social distancing practice and associated factors in response to COVID-19 pandemic at West Guji Zone, Southern Ethiopia, 2021: A community based cross-sectional study

**DOI:** 10.1371/journal.pone.0261065

**Published:** 2021-12-20

**Authors:** Anteneh Fikrie, Elias Amaje, Wako Golicha

**Affiliations:** School of Public Health, Institute of Health, Bule Hora University, Bule Hora, Ethiopia; China University of Mining and Technology, CHINA

## Abstract

**Background:**

Curtailing physical contact between individuals reduces transmission and spread of the disease. Social distancing is an accepted and effective strategy to delay the disease spread and reduce the magnitude of outbreaks of pandemic COVID-19. However, no study quantified social distancing practice and associated factors in the current study area. Therefore, the study aimed to assess social distancing practice and associated factors in response to COVID-19 pandemic in West Guji Zone, Southern Ethiopia, 2020.

**Methods and materials:**

A Community based cross-sectional study design was conducted among randomly selected 410 household members of Bule Hora Town, West Guji Zone. Data were collected by pre-tested interviewer administered structured questionnaire adapted from previous peer reviewed articles. The data were coded and entered in to Epi data version 3.5 and analyzed by SPSS version 23. The bivariate and multivariate logistic regressions analysis was done to identify factors associated with social distancing practice. Adjusted odds ratio with 95% confidence interval and p value <0.05 were used to declare statistical significance.

**Result:**

Out of 447 planned samples, 410 participants were successfully interviewed and included into final analysis; making the response rate of 91.7%. The median (±IQR) age of study participants was 28(±9) years. In this study, 38.3% [95% CI: 33.5%, 43.1%)] of the study participants have good social distancing practices for the prevention of COVID-19. Age group 26–30 years [AOR = 2.56(95% CI: 1.18–5.54)] and 31–35 years [AOR = 3.57(95%CI: 1.56–8.18)], employed [AOR = 6.10(95%CI: 3.46–10.74)],poor knowledge [AOR = 0.59 (95% CI:0.36–0.95)], negative attitude [AOR = 0.55 (95% CI:0.31–0.95)] and low perceived susceptibility [AOR = 0.33(95%CI: 0.20–0.54)] were significantly associated with good social distancing practice.

**Conclusion:**

Social distancing practice is relatively poor in the study area. The knowledge and attitude level of participants were identified to be the major factors for the observed poor social distancing practice. Sustained efforts to improve awareness and attitudes towards COVID-19 prevention might improve adherence to social distancing practices.

## Introduction

Corona virus Disease 2019 (COVID -19) caused by the novel coronavirus (SARS CoV-2) has posed a public health emergency and a global crisis rapidly as of December 2019 originated in Wuhan, a city in the Hubei Province of China [1]. The viruses are a large family of viruses that cause illnesses ranging from common cold to more severe diseases such as Middle East respiratory syndrome (MERS-CoV) and severe acute respiratory syndrome (SARS-CoV-1). SARS-CoV -2 is a novel coronavirus that has not been previously identified in humans [2]. As of now, the source of the outbreak is unknown with certainty. However, it is believed that the virus might have link with a wet market (i.e. seafood’s and live animals) from the Wuhan city [2,3]. The important mode of virus transmission is via person-to-person occurring mainly via respiratory droplets, and by contact with contaminated surfaces [1,4]. According to the World Health Organization (WHO), symptoms of infection with the virus include fever, cough, and shortness of breath and breathing difficulties. Severe infection can lead to pneumonia, multiple organ failure and even death [5].

The World Health Organization (WHO) has declared COVID-19 as a pandemic on 12th March 2020 [6]. As of 1 December, more than 254 million cases and 5.1 million deaths have been reported globally until 15 November 2021. The highest burden of the disease is in WHO American region with more than 95, 120, 017 (37.4%) confirmed cases recorded so far, whereas the lowest record 6, 186, 377 (2.4%) and 9, 794, 363(3.5%) of cases reported from WHO African and Western Pacific region respectively [7]. In Africa, the numbers of COVID-19 cases and impacted countries have been increasing steadily and there are no virus free countries in the region. Thus, South Africa, Kenya, and Ethiopia report higher number of new cases [8]. In Ethiopia 368,822 cases and 6,623 deaths were reported until 15 November 2021 [7]. However, reported statistics is likely to represent an underestimation of the true burden of the disease owing to shortcomings in active surveillance and diagnostic capacity of the country [9]. Across the globe, countries have been implementing different disease control and prevention measures to combat the pandemic with the objective of slowing disease transmission and reducing associated morbidity and mortality [6]. These measures include case identification, testing, isolation and care for all cases, tracing and quarantine of all contacts, social distancing at individual and community levels [10]. Moreover, SARS-CoV-2 has affected several countries across the globe, prompting governments to impose social distancing measures to slow the spread of infection. Ethiopia is also implementing the social distancing measures to reduce the spread of the virus [11].

Social distancing is one category of non-pharmaceutical interventions (NPI) which means making changes in our everyday routines in order to minimize close contact with others, including: avoiding crowded places and non-essential gatherings, avoiding common greetings, such as handshakes, limiting contact with people at higher risk (e.g. older adults and those in poor health), keeping a distance of approximately 2 meters from others to reduce risk of infection [12,13]. Staying at least six feet (i.e. 2 meter) away from other people reduces risk of acquiring COVID-19 [14]. Evidence from past influenza pandemics revealed that social distancing practice reduces spread of the virus [15,16]. A study aimed at identifying whether controlling epidemic spread by social distancing do it well or not at all concluded that social distancing is the most cost-effective strategy of controlling the epidemic [17]. Particularly, during the early phase of the pandemic where neither proven treatment nor vaccination is available, implementation of non-pharmaceutical interventions (NPIs) like social distancing is an effective and option [18].

Social distancing changes the behavior of an individual that prevent disease transmission by reducing contact rates, but the benefits depend on the extent to which it is practiced by individuals. In the absence of other intervention measures, optimal social distancing reduces the risk by 30% [19]. On the other hand, according to European Centre for Disease Prevention and Control technical report it was estimated that if social distancing had been conducted one week, two weeks, or three weeks earlier in China, the number of COVID-19 cases could have been halted by 66%, 86%, and 95%, respectively [12]. A study conducted among government employees in Ethiopia revealed that, more than nine-in-ten (94.8%) avoids handshaking; whereas 89.5% and 88.1% practiced physical distancing and avoided mass gatherings and crowded places respectively [20]. Another study conducted in the same area found that the majority of respondents had reflected good knowledge, positive attitude and low magnitude of practice regarding COVID-19 prevention activities [21]. A nationwide online cross-sectional survey conducted in Uganda found that 14.7% of participants were not practicing social distancing [22].

The study was conducted after the government underwent series of lockdown as a major means of limiting the spread disease. So this would reveal whether the communities are adhered or not to COVID-19 prevention measures particularly of the social distancing practices. More importantly, in resource constrained countries like Ethiopia, social distancing is an effective and affordable way of containing the pandemic. However, few are known extent to which individuals adhere to recommended social distancing practice and factors associated with it. Therefore, this study aimed to assess social distancing practice and associated factors in response to COVID-19 in Bule Hora town, southern Ethiopia 2020.

## Materials and methods

### Study setting, *design and period*

A community based cross-sectional study was conducted in Bule Hora town, West Guji Zone, Oromia Region from September 15–30, 2020. Bule Hora town is the capital of West Guji Zone, located 467 km South to Addis Ababa, Ethiopia’s capital. Administratively the town has 4 kebeles (i.e. Smallest administrative unit in Ethiopia). The estimated number of the households in the town is 11, 766.

#### Study population, sample size determination and procedure

All households of Bule Hora town were source population. Whereas, all randomly selected households within the town during the data collection period were study population. All adult household members aged 18 years and above were included in the study. Critically ill and adults who lived less than six months in the town were excluded from the study. The sample size for the first objective was determined by using single population proportion formula. Considering the proportion of social distancing of 35.3% obtained from previous study conducted in Bangladesh [23], 95% confidence interval (Z = 1.96) and 5% margin of error (d). Then, by substituting the aforementioned figure in to the single population proportion sample size calculation formula, the calculated sample size became 351. Sample size for second objective (identification of factors associated with social distancing practice) was computed by Epi info7 Statcalc version 7.1.4.0 software with the assumptions of, 95% level of confidence, power of 80%, the ratio of exposed to unexposed 1:1 and percent of outcome in unexposed group 64.2% and AOR of 1.9. The percent of outcome in unexposed group and AOR were taken from the study conducted in UK [24]; the determinate variable was (Age 18–34 years). Then required sample for the second objective became 406. Therefore among the two sample sizes calculated, the largest sample size was obtained from the second objective. Then after adding 10% non-response rate to 406, the final minimum total sample size became 447. First all the four kebeles (lowest administrative unit) of the town administration were included in the study. Then after getting the number of households from the town administration Office, the calculated sample size were allocated proportional to the size of population in each kebele. Subsequently, Simple random sampling technique was used to select the households from each kebele. Within selected households, adults (at least 18 years) old were interviewed. In case of presence of more than one eligible adult in the household, lottery method was used to select one adult for the interview.

#### Data collection procedures and quality assurance

The data were collected by a pretested structured interviewer-administered questionnaire. The questions which assess the level of compliance to social distancing practice and associated factors were adapted from previous peer reviewed articles, WHO and FMOH guidelines [10,11,23–25]. The adapted questions were modified and contextualized to fit the local situation and the research objectives. Primarily the questionnaire was prepared in English **(S1 File)** and then translated to the local language “Afaan Oromo” by fluent speakers of both language and then translated back to English to keep the consistency of the questionnaire. The questionnaire contains socio-demographic characteristics, chronic medical conditions, risk perceptions towards COVID-19, knowledge and attitude towards social distancing practices for the prevention of COVID 19, Social distancing practice related questions.

The knowledge level about social distancing practice and COVID-19 was assessed using “Yes’ or “No” questions. Five point Likert scale was used to assess attitude (5 = Strongly Agree, 4 = agree, 3 = neutral, 2 = disagree and 1 = strongly disagree) related to social distancing and COVID-19. Whereas, a three point Likert scale was used to assess social distancing practice of the participants (2 = Always, 1 = Occasional, 0 = Never). Two days training was given for data collectors and supervisors on data collection tools and procedures. During data collection personal protective equipment like sanitizer, face mask and glove were secured for each data collectors and supervisors. Questionnaire was pretested on 5% of expected sample size (n = 22) at Gerba town, one week prior to data collection to check whether the questionnaire was accurate. No adjustment was necessary. The overall supervision was carried out by investigators during data collection period on daily basis and data were cleared and checked daily its completeness and consistency before processing and analysis. During data collection a participant having clinical features related to COVID-19 were screened by digital thermometer. But no one has been identified with high grade fever. All the study participants were encouraged to participate in the study voluntarily and at the same time they were also told that they have the right not to participate.

### Study variables and operational definition

The dependent variable of the study was social distancing practice and the explanatory variables were **Socio-demographic factors (**sex, age, residence, income, religion, educational status, marital status, occupation, household tenure and family size), **knowledge** and **attitude** towards social distancing for the responses of COVID-19, **Risk perceptions** towards COVID-19 and **Chronic medical history.**

- **Knowledge:** Participants who answered ≥50% of correct answers among the total knowledge related questions were classified as having a good knowledge. Whereas participants who answered < 50% of the questions were classified as having poor knowledge.
- **Attitude:** Participants who answered ≥50% of correct answers among the total attitude related questions were classified as having a positive attitude. Whereas, participants who answered <50% of questions were classified as having negative attitude.
- **Social distancing practice:** Eight questions with a three Likert scale were collected and the total social distancing practice score was calculated by summing the Likert score. Thus, **p**articipants who answered ≥50% of correct answers among the total **eight** social distancing practice related questions were regarded as having ***good practice***. Whereas, participants who answered less than 50% of the questions were taken as having a ***poor practice*.**
- **Perceived susceptibility:** is how likely one considered oneself (his/her families) would be infected with COVID-19 if no preventive measure was taken. Hence, Participants who scored ≥50% of questions were categorized as having high perceived susceptibility. Whereas, participants who scored <50% of questions were categorized as having low perceived susceptibility of contracting COVID-19.
- **Perceived seriousness:** is perceived chance of having COVID-19 cure and survival if infected with COVID-19. Participants who scored ≥50% of questions were categorized as having high perceived severity. Whereas, participants who scored <50% of questions were categorized as having low perceived severity of COVID-19.
- **Perceived self-efficacy:** A person’s belief in his or her ability to practice social distancing practice. Participants who scored ≥50% of questions were categorized as having high perceived self-efficacy. Whereas, participants who scored <50% of questions were categorized as having low perceived self-efficacy of practicing social distancing.
- **Perceived Benefits:** is perceived benefits of practicing social distancing for the prevention of COVID-19. Participants who scored ≥50% of questions were categorized as having high perceived benefits from practicing social distancing. Whereas, participants who scored <50% of questions were categorized as having low perceived benefits of practicing social distancing.
- **Perceived Barriers**: Perceived barriers to social distancing practice as a preventive measure of COVID-19. Participants who scored ≥50% of questions were categorized as having high perceived barriers. Whereas, participants who scored <50% of questions were categorized as having low perceived barriers to measures of social distancing practice.

#### Data processing and analysis

The collected data were cleaned, coded, and entered by Epi-DATA version 3.5 and exported to statistical package for social science (SPSS) version 23.0 for analysis. Median with Inter quartile range (IQR) was used to summarize quantitative variable. The results were presented by tables, figures and different interactive charts. Binary logistic regression analysis was done to examine statistical association between social distancing practices and independent variables. Variables with p-value <0.25on bivariate analysis were further entered into multivariable logistic regression to identify statistically significant variables. The multicollinearity between independent variables was checked by using variation inflation factor (VIF) and tolerance test. The Hosmer-Lemeshow test was done to check the model fitness for analysis. A reliability analysis of the questionnaires was checked and Cronbach’s alpha showed the questionnaire were passed the acceptable reliability number (α = 0.82). Adjusted odds ratios (AOR) together with 95% CI were used to estimate the strength of associations and statistical significance was declared at a p-value < 0.05 (S2 File).

#### Ethical considerations

Primarily the study protocol was officially approved by the Research and Publication Directorate of Bule Hora University (Ref.No: BHU/RPD/270/13). Based on the approval, an official letter was written by RPD to Bule Hora Town Health office and Bule Hora Town Administration office. The Town health office wrote the letter to respective kebeles for cooperation. At last the data were collected after assuring the confidentiality nature of responses and obtaining verbal consent from the study participant.

## Results

### Socio-demographic characteristics of study participants

Out of the total of 447 sampled participants 410 of them were voluntarily interviewed and make the response rate of 91.7%. The median (±IQR) age of study participants was 28(±9) years of age. The majority, 129(31.5%) of study participants were found in the age group of 26–30 years. More than half, 223 (54.4%) of the participants were female. Likewise, nearly, three-fourth of the participants was married. About 142 (34.6%) of the participants have no formal education. Nearly one-fifth, 92 (22.4) of the study participants were government employed. Two hundred seven, (50.5%) and 132(32.3%) of participants have TV and Radio respectively. Concerning housing tenure, 252 (61.5%) of respondents were living in rental houses. Pertaining the family size more than two-fifth, 176 (43%) of the participants have a family size of ≥5. The majority of the study participants, 146 (35.6%) have a monthly income of ≤1000 (Table 1).

**Table 1:** Socio-demographic characteristics of study participants at Bule Hora town, 2020.

Variable	Category	Frequency	Percent %
Age	≤20	46	11.2
	21–25	92	22.4
	26–30	129	31.5
	31–35	78	19
	>35	65	15.9
Sex of respondent	Male	187	45.6
	Female	223	54.4
Marital status	Married	304	74.1
	Single	84	20.5
	Divorced	11	2.7
	Widowed	11	2.7
Educational status	No formal education	142	34.6
	Primary completed	50	12.2
	Secondary completed	112	27.3
	Higher and above	106	25.9
Occupational status	Government employed	92	22.4
	Merchant/Trade	76	18.5
	Farmer	37	9
	Private	79	19.3
	Housewife	90	22
	Others	36	8.8
Housing tenure	Private	158	38.5
	Rental	252	61.5
Television	Yes	207	50.5
	No	203	49.5
Radio	Yes	132	32.3
	No	277	67.7
Family size	≤2	78	19
	3–4	156	38
	≥5	176	43
Monthly income of respondents in Ethiopian birrs*	≤1000	146	35.6
	1001–3000	132	32.2
	3001–5000	63	15.4
	≥5001	69	16.8

*1 Birr = 0.0229$.

### Chronic medical condition and behavior of the study participants

Regarding the participant’s chronic medical condition and behavioral history about quarter, 99(24.1%) of the study participants have at least one type of chronic medical history. Twenty five (6.1%), 32 (7.8%) and 24 (5.9%) of participants have DM, hypertension and asthmatic problems respectively. On the other hand small proportion, 28(6.8%) of the study participants are smoke cigarette (Table 2).

**Table 2:** Medical condition of the study participants at Bule Hora town, 2020.

Variable	Category	Frequency	Percent %
Do you have DM	Yes	25	6.1
	No	288	70.2
	I don’t know	97	23.7
Do you have HTN	Yes	32	7.8
	No	286	69.8
	I don’t know	92	22.4
Do you have Cardiac problem	Yes	9	2.2
	No	313	76.3
	I don’t know	88	21.5
Do you have Asthma	Yes	24	5.9
	No	318	77.6
	I don’t know	68	16.6
Do you have Cancer	Yes	1	0.2
	No	318	77.6
	I don’t know	91	22.2
Do you have HIV/AIDS	Yes	28	6.8
	No	247	60.2
	I don’t know	135	32.9
Do you smoke cigarette	Yes	28	6.8
	No	382	93.2

### Risk perceptions of the study participants about social distancing practice perceived susceptibility

Of the total more than half, 232 (56.6%) of the respondents were strongly disagreed that there is less chance to transmit infection to family members from sick person (Table 3).

**Table 3:** Perceived susceptibility of study participants toward COVID-19 pandemic.

Perceived susceptibility	Responses				
	S. disagree No (%)	Disagree No (%)	Neutral No (%)	Agree No (%)	S. agree No (%)
Less chance to transmit infection to family members from sick person?	232 (56.6)	96 (23.4)	26(6.3)	42(10.2)	14(3.4)
No chance to get infection for healthy person	157(38.3)	157(38.3)	34(8.3)	46(11.2)	16(3.9)
Little chance to get infection for young	128(31.2)	178(43.4)	46(11.2)	40(9.8)	18(4.4)
High chance to get infection from foreigner	81(19.8)	122(29.8)	70(17.1)	108(26.3)	29(7.1)
Easily get disease in crowded place	50(12.2)	94(22.9)	61(14.9)	165(40.2)	40(9.8)
Healthy life style will reduce the chance of infection	39(9.5)	101(24.6)	61(14.9)	163(39.8)	46(11.2)

#### Perceived severity

Out of the total respondents, 148(36.1%) agreed that COVID-19 will be more serious among elderly and people with comorbidities. Majority of the respondents agreed that if they were infected with COVID-19, they will suffer severe symptoms (Table 4).

**Table 4:** Perceived severity of study participants toward COVID-19 pandemic.

Perceived severity	Responses				
	S. disagree No (%)	Disagree No (%)	Neutral No (%)	Agree No (%)	S. agree No (%)
COVID-19 will be more serious among elderly and people with comorbidities?	56(13.7)	54(13.2)	50(12.2)	148(36.1)	102(24.9)
If I were infected with COVID-19, I will suffer severe symptoms	51(12.4)	42(10.2)	67(16.3)	180(43.9)	70(17.1)
If I were infected with COVID-19, I could not survive	50(12.2)	68(16.6)	101(24.6)	120(29.3)	70(17.1)
I can suffer from COVID-19 without signs and symptoms	49(12)	74(18)	100(24.4)	137(33.4)	48(11.7)
COVID-19 will be treated if I were infected	35(8.5)	67(16.3)	102(24.9)	151(36.8)	55(13.4)
If I were infected with COVID-19, i will recover spontaneously	40(9.8)	78(19)	99(24.1)	125(30.5)	68(16.6)

#### Perceived self-efficacy

Out of the total respondents, 123(30%) were much confident that they can get access to the reliable health information on COVID-19. About one hundred nineteen (29%) of respondents were much confident that they will eat healthy diet to prevent covid-19(Table 5).

**Table 5:** Perceived self-efficacy of study participants toward COVID-19 pandemic.

Perceived self-efficacy	Responses				
	No No (%)	Low confident No (%)	Neutral No (%)	Much No (%)	High confident No (%)
I can get access to the reliable health information on COVID-19	84(20.5)	95(23.2)	30(7.3)	123(30)	78(19)
I will eat healthy diet to prevent COVID-19	88(21.5)	58(14.1)	43(10.5)	119(29)	102(24.9)
To prevent COVID-19, I will wash my hands	61(14.9)	58(14.1)	26(6.3)	130(31.7)	135(32.9)
I can prevent COVID-19	39(9.5)	66(16.1)	75(18.3)	148(36.1)	82(20)
To prevent COVID-19, I will avoid visiting crowded places	42(10.2)	71(17.3)	46(11.2)	160(39)	91(22.2)
To prevent COVID-19, I will use face mask whenever I go to crowded place	40(9.8)	62(15.1)	51(12.4)	154(37.6)	103(25.1)

#### Perceived barriers

Concerning the perceived barriers of respondents, about one-third 136(33.2) of them were strongly agreed that it is hard refraining social gatherings at one’s home. One hundred fifty one (36.8%) of respondents were strongly agreed that it is hard to stay home too much (Table 6).

**Table 6:** Perceived barriers of study participants toward COVID-19 pandemic.

Perceived barriers	Responses				
	S. disagree No (%)	Disagree No (%)	Neutral No (%)	Agree No (%)	S. agree No (%)
Hard to refrain social gatherings at home	84(20.5)	42(10.5)	26(6.3)	122(29.8)	136(33.2)
Hard to stay home too much	49(12)	50(12.2)	24(5.9)	136(33.2)	151(36.8)
Difficult using face mask daily?	41(10)	57(13.9)	25(6.1)	140(34.1)	147(35.9)
Can’t afford to buy soap/alcohol containing hand sanitizer	27(6.6)	46(11.2)	60(14.6)	156(38)	120(29.3)

#### Perceived benefits

Concerning the perceived benefits of respondents, about 191(46.6%) of them were agreed that doing protective measures of covid-19 is caring for themselves and their families. Nearly one-third, 142(34.6%) of respondents were greed that keeping social distancing is setting good example for others19 (Table 7).

**Table 7:** Perceived benefits of study participants toward COVID-19 pandemic.

Perceived benefits	Responses				
	S. disagree No (%)	Disagree No (%)	Neutral No (%)	Agree No (%)	S. agree No (%)
When I am doing something protective measures of COVID-19, I am caring for myself and my families	73(17.8)	31(7.6)	43(10.5)	191(46.6)	72(17.6)
When I keep social distancing, I am setting a good example for others	37(9)	75(18.3)	73(17.8)	142(34.6)	83(20.2)
When I wear face mask at crowded area, I am decreasing my chances of contracting COVID-19?	28(6.8)	73(17.8)	72(17.6)	159(38.8)	78(19)
Staying home will reduce my chances of contracting COVID-19?	24(5.9)	54(13.2)	76(18.5)	155(37.8)	101(24.6)

### Study participants’ knowledge about risky groups, symptoms, prevention methods of COVID-19

Overall, 222(54.1%) [95% CI (49.3, 59.2%)] of the study participants have good knowledge towards COVID-19 and its prevention methods. Four hundred eight, (99.5%) of respondents ever heard about corona virus. Majority, 157 (38.5%) of respondents obtained information regarding COVID-19 from health personnel. One hundred sixty six (40.5%) of participants claimed that health personnel is trusted source of information. More than half, 246 (60%) of respondents mentioned that the main causes of COVID-19 is virus (Table 8).

**Table 8:** Participants’ knowledge of risky groups, symptoms, prevention methods of COVID-19 among Bule Hora town adults (n = 410).

Variable	Category	Frequency	Percent
Ever heard about corona virus	Yes	98	99.5
	No	2	0.5
What was your source of information?	Health personnel	157	38.3
	Social media	76	18.5
	FMOH sources	23	5.6
	Mass media	125	30.5
	Friends/family members/relatives	29	7.1
Trusted source of information	Health personnel	166	40.5
	Social media	58	14.1
	FMOH sources	38	9.3
	Mass media	128	31.2
	Friends/family members/relatives	20	4.9
The cause of COVID-19 is?	Virus	246	60
	Others*	164	40
Can COVID-19 transmit human-to-human?	Yes	386	94.1
	No	24	5.9
What are the modes of transmission of COVID-19?	Airborne	152	37.1
	Physical contact with contaminated object	208	50.7
	Physical contact with infected people	154	37.6
	Eating raw meat	148	36.1
Prevention methods of COVID-19	Avoid close contact with people who are sick	237	57.8
	Frequent hand washing with soap and water/alcohol-based hand sanitizer	322	78.5
	Avoid touching your eye, nose, mouth with unwashed hands	169	41.2
	Avoid shaking hands	152	37.1
	Avoid crowded place	255	62.6
	Disinfecting/cleaning objects and surfaces	134	32.7
	Stay at home/work at home	84	20.5
	Practicing good respiratory hygiene	113	27.6
The main clinical symptoms of COVID-19	Fever	344	83.9
	Dry Cough	318	77.6
	Breathing difficulty	182	44.4
	Fatigue	110	26.8
	Sneezing	199	48.5
	Headache	163	39.8
There is no effective vaccine for COVID-19?	Yes	233	56.8
	No	177	43.2
There is no any definitive treatment of COVID-19 currently?	Yes	278	67.8
	No	132	32.2
High-risk population of COVID-19	Children	128	31.2
	Elderly	179	43.7
	Pregnant women	72	17.6
	People with chronic disease	121	29.5
	Cigarette smokers	43	10.5

### Attitudes of study participants about COVID-19 and social distancing

Out of the total respondents, 298(72.7% [95% CI (68.8, 76.6%)] of the study participants have positive attitude towards the social distancing practices for the prevention of COVID-19. Of the total study participants, 234(57.1%) were strongly disagreed to stay at home for certain period (14 days) to prevent covid-19 spread if government will order so. Nearly one-third, 125(30.5%) of the respondents were agreed that social distancing can prevent covid-19 spread (Table 9).

**Table 9:** Attitudes of study participants about COVID-19 and social distancing.

Questions	Responses				
	S. disagree No (%)	Disagree No (%)	Neutral No (%)	Agree No (%)	S. agree No (%)
Do you like to stay at home for certain period (14 days) to prevent COVID-19 spread if government will order so?	234(57.1)	68(16.6)	25(6.1)	66(16.1)	17(4.1)
Do you think that social distancing (e.g. stay 2 m apart, avoiding crowds, etc.) can prevent COVID-19 spread?	69(16.8)	101(24.6)	82(20)	125(30.5)	33(8)
Do you agree that we should cancel business/recreational trips at this time?	69(16.8)	149(36.3)	101(24.6)	60(14.6)	31(7.6)
Do you believe that working from home can help to control COVID-19?	68(16.6)	109(26.6)	120(29.3)	67(16.3)	46(11.2)
When someone has signs and symptoms of COVID-19, I can confidently keep my physical distance from him/her?	33(8)	43(10.5)	75(18.3)	184(44.9)	75(18.3)
Do you think that, Ethiopia is in a good position to **contain** COVID-19?	28(6.8)	80(19.5)	85(20.7)	130(31.7)	87(21.2)

### Social distancing practice of study participant for COVID-19 prevention

In this study nearly two-in-five, 157 (38.3%) [95% CI (33.5, 43.1%)] of the study participants have good social distancing practices for the prevention of COVID-19. Out of total respondents, 169(41.2%) always avoided contact with someone who is displaying symptoms of coronavirus. Two hundred fifty six, (62.4%) of respondents never avoided non-essential use of public transport when possible. Majority, 278(67.8%) of respondents never work at home (Table 10).

**Table 10:** Social distancing practice of study participant for COVID-19 prevention.

Questions	Responses		
	Always No (%)	Occasional No (%)	Never No (%)
Avoid contact with someone who is displaying symptoms of coronavirus	169(41.2)	154(37.6)	87(21.2)
Avoid non-essential use of public transport when possible	132(32.2)	22(5.4)	256(62.4)
Work at home	121(29.5)	11(2.7)	278(67.8)
Avoid large and small gatherings in public spaces (pubs, restaurants, leisure centers)	163(39.8)	7(1.7)	240(58.5)
Avoid gatherings with friends and family	135(32.9)	19(4.6)	256(62.4)
Maintaining non-contact greetings	348(84.9)	22(5.4)	40(9.8)
Maintain 2 meters distance between yourself & other people	156(38)	40(9.8)	214(52.2)
Stay home when ill	91(22.2)	88(21.5)	231(56.3)

### Factors associated with knowledge level of respondents towards COVID-19

The out puts of the bi-variable and multivariable logistic regression analyses of factors associated with knowledge level of the participant’s found that, being employed were 65% more likely to have good knowledge regarding the prevention measures of COVID-19 compared to unemployed respondents **[AOR =** 1.65(1.05–2.58)]. Similarly, respondents who had positive attitude were 65% more likely of having a good knowledgeable than respondents who had negative attitude [AOR = 1.65(1.02–2.66)]. Respondents who had low perceived susceptibility were 35% less likely to have good knowledge than their counter part [AOR = 0.65(0.43–0.99)] (Table 11).

**Table 11:** Factors associated with knowledge of risky groups, symptoms, and prevention methods of COVID-19 among households of Bule Hora town, Southern Ethiopia, 2020.

Variables		Knowledge				COR (95% Cl)	AOR (95% Cl)
		Good		Poor			
		No	%	No	%		
Sex of respondent							
	Male	109	58.3	78	41.7	1.36 (0.91–2.01)	1.25 (0.82–1.90)
	Female	113	50.7	110	49.3	1	1
Age of respondents							
	**≤**20	21	45.7	25	54.3	0.63(0.27–1.35)	
	21–25	48	52.2	44	47.8	0.82(0.43–1.56)	
	26–30	69	53.5	60	46.5	0.87(0.47–1.58)	
	31–35	47	60.3	31	39.7	1.14(0.58–2.23)	
	>35	37	56.9	28	43.1	1	
Educational status							
	No formal education	76	53.5	66	46.5	1	
	Primary completed	25	50	25	50	0.86(0.45–1.65)	
	Secondary completed	61	54.5	51	45.5	1.03(0.63–1.70)	
	College and above	60	56.6	46	43.4	1.13(0.68–1.87)	
Occupational status							
	Employed^#^	81	62.8	48	37.2	1.67(1.09–2.56)	1.65(1.05–2.58)**
	Unemployed	141	49.8	140	50.2	1	1
TV							
	Yes	118	57	89	43	1	1
	No	104	51.2	99	48.8	0.79(0.53–1.16)	0.86(0.57–1.30)
Radio								
	Yes	77	58.3	55	41.7	1	
	No	144	52	133	48	0.77(0.50–1.17)	
Family size								
	≤2	38	47.7	40	42.3	0.67(0.39–1.15)	0.71(0.41–1.26)
	3–4	81	51.9	75	48.1	0.76(0.49–1.18)	0.77(0.48–1.21)
	≥5	103	58.5	73	41.5	1	1
At least one Chronic disease								
	Yes	60	60.6	39	39.4	1	1
	No	162	52.1	49	47.9	0.70(0.44–1.12)	0.80(0.48–1.34)
Attitude							
	Negative	51	45.5	61	54.5	1	1
	Positive	171	57.4	127	42.6	1.16(1.04–2.49)	1.65(1.02–2.66)*
Perceived Susceptibility							
	Low susceptibility	83	48.5	88	51.5	0.67(0.45–1.07)	0.65(0.43–0.99)*
	Highly susceptibility	139	58.2	100	41.8	1	1
Perceived Severity							
	Less severe	45	57	34	43	1.17(0.71–1.92)	
	Highly Severe	174	53	154	47	1	
Perceived self-efficacy							
	Low self-efficacy	63	57.8	46	42.2	1.23(0.78–1.90)	
	High self-efficacy	159	52.8	142	47.2	1	
Perceived Benefits							
	Not Benefits	26	45.6	31	54.4	0.67(0.38–1.17)	
	Benefits	196	55.5	157	44.5	1	

*p-value <0.05,

** p-value <0.001,

***p-value<0.0001;

# Government and private employed.

### Factors associated with attitudes of study participants about COVID-19 and social distancing

Bi-variable and multivariable binary logistic regression was used to identify factors associated with the attitude of study participants regarding COVID-19 and social distancing practices. Accordingly, variables which had a p-value of ≤0.25 during bivariable logistic regression were further entered to multivariable binary logistic regression. After adjusting for confounding variables, the odds of positive attitude was 68% [AOR = 0.32(0.13–0.80)] and 66% [AOR = 0.34(0.14–0.82)] reduced among respondents who were in age group of 26–30 and 31–35 years as compared to respondents who were above 35 years of age respectively. Respondents who had perceived less severity and perceived low self-efficacy were 43% [AOR = 0.57(0.32–0.99)] and 48% [AOR = 0.52(0.31–0.88)] less likely to have positive attitude than their counter parts respectively (Table 12).

**Table 12:** Factors associated with attitudes of study participants about COVID-19 and social distancing among Households of Bule Hora town, Southern Ethiopia, 2020.

Variables		Attitude				COR (95% Cl)	AOR (95% Cl)
		Positive		Negative			
		No	%	No	%		
Sex of respondent							
	Male	137	73.3	50	26.7	1.05 (0.68–1.63)	
	Female	161	72.2	62	27.8	1	
Age of respondents							
	**≤**20	34	73.9	12	26.1	0.57(0.22–1.45)	0.41(0.12–1.33)
	21–25	67	72.8	25	27.2	0.54(0.24–1.20)	0.43(0.15–1.20)
	26–30	91	70.5	38	29.5	0.48(0.23–1.03)	0.32(0.13–0.80)*
	31–35	52	66.7	26	33.3	0.40(0.18–0.90)	0.34(0.14–0.82)*
	>35	54	83.1	11	16.9	1	1
Educational status							
	No formal education	101	71.1	41	28.9	1	1
	Primary completed	40	80	10	20	1.64(0.74–3.55)	1.73(0.73–4.11)
	Secondary completed	78	69.6	34	30.4	0.93(0.54–1.60)	1.27(0.67–2.41)
	College and above	79	74.5	27	23.5	1.18(0.67–2.09)	1.56(0.83–2.92)
Occupational status							
	Employed^#^	89	69	40	31	0.76(0.48–1.21)	
	Unemployed	209	25.6	72	74.4	1	
Family size							
	≤2	47	60.3	31	39.7	0.55(0.31–0.97)	0.70(0.33–1.50)
	3–4	122	78.2	34	21.8	1.30 (0.78–2.16)	1.63(0.87–3.06)
	≥5	129	73.3	47	26.7	1	1
At least one Chronic disease							
	Yes	71	71.7	28	28.3	1	
	No	227	73	84	27	1.06(0.64–1.76)	
Perceived Susceptibility							
	Low susceptibility	119	69.6	52	30.4	0.76(0.49–1.18)	1.06(0.65–1.74)
	Highly susceptibility	179	74.9	60	23.1	1	1
Perceived Severity							
	Less severe	46	58.2	33	41.8	0.44(0.26–0.73)	0.57(0.32–0.99)*
	Highly Severe	249	75.9	79	24.1	1	1
Perceived self-efficacy							
	Low self-efficacy	68	62.4	41	37.6	0.51(0.32–0.81)	0.52(0.31–0.88)*
	High self-efficacy	230	76.4	71	23.6	1	1
Perceived Barriers							
	Barriers	47	68.1	22	31.9	0.76(0.43–1.34)	
	Not barriers	251	73.6	90	26.4	1	

*p-value <0.05,

** p-value <0.001,

***p-value<0.0001;

# Government and private employed.

### Factors associated with social distancing practice for the prevention of Covid-19

During the bivariable binary logistic regression, age of respondents, educational status, having Television, Cigarette smoking, Attitude level, knowledge status, Perceived Susceptibility, Perceived Barriers and Perceived Benefits were statistically significant at a p-value of <0.25 and identified as the candidates for the multivariable binary logistic regression analysis so as to control the potential presence of confounding variables. As a result, at the multivariate model the Age of respondents, occupational status, knowledge status, attitude level and perceived susceptibility were significantly associated with good social distancing practice at p<0.05.

Accordingly, the odds of good social distancing practice was 45% reduced among the household who have negative attitude towards social distancing practices for the prevention of COVID-19 as compared to their counter parts [AOR = 0.55 (95% CI:0.31–0.95)]. Similarly, the odds of good social distancing practices was 41% reduced among the household who have poor knowledge about social distancing practices as compared to their counter parts [AOR = 0.59 (95% CI:0.36–0.95)]. On the other hand, the odds of good social distancing practices was 67% reduced among individuals who have low susceptibility perception for contracting COVID-19 as compared to individuals who have high susceptibility perception of contracting COVID-19 [AOR = 0.33(95%CI: 0.20–0.54)]. Those respondents who were employed were 6 times more likely to comply with social distancing practice as compared to those who were unemployed [AOR = 6.10(95%CI: 3.46–10.74)]. The age of respondents was also positively associated with social distancing practices. The odd of good social distancing practices was 2.5 [AOR = 2.56(95% CI: 1.18–5.54)] and 3.5 [AOR = 3.57(95%CI: 1.56–8.18)]times higher among individuals who are in the age group of 26–30 and 31–35 years as compared to individuals who are above 35 years of age respectively (Table 13).

**Table 13:** Factors associated with social distancing practices for the prevention of COVID-19 among households of Bule Hora town, Southern Ethiopia, 2020.

Variables		Practice				COR (95% Cl)	AOR (95% Cl)
		Good		Poor			
		No.	(%)	No.	(%)		
Sex of respondent							
	Male	73	46.5	114	45.1	0.94 (0.63–1.40)	
	Female	84	53.5	139	54.9	1	
Age of respondents							
	**≤**20	16	10.2	30	11.9	1.50(0.66–3.42)	2.26(0.81–6.34)
	21–25	36	22.9	56	22.1	1.81(0.90–3.63)	2.04(0.87–4.77)
	26–30	51	32.5	78	30.8	1.84(0.95–3.55)	2.56(1.18–5.54)*
	31–35	37	23.6	41	16.2	2.54(1.25–5.18)	3.57(1.56–8.18)**
	>35	17	10.8	48	19.0	1	1
Educational status							
	No formal education	45	28.7	97	38.3	1	1
	Primary completed	23	14.6	27	10.7	1.83(0.95–3.54)	1.80(0.81–4.00)
	Secondary completed	43	27.4	69	27.3	1.34(0.79–2.25)	1.45(0.76–2.97)
	College and above	46	29.3	60	23.7	1.65(0.98–2.78)	0.65(0.33–1.28)
Occupational status							
	Employed^#^	80	51.8	49	19.4	4.32(2.78–6.72)	6.10(3.46–10.74)***
	Unemployed	107	68.2	174	68.8	1	1
Housing tenure							
	Private	58	36.9	100	39.5	1	
	Rental	99	63.1	153	60.5	1.11(0.74–1.68)	
TV							
	Yes	86	54.8	121	47.8	1	1
	No	71	45.2	132	52.2	0.75(0.50–1.12)	0.65(0.40–1.05)
Radio							
	Yes	54	34.4	78	31	1	
	No	103	65.6	174	69	0.85(0.56–1.30)	
Family size							
	≤2	27	17.2	51	20.2	0.88(0.50–1.54)	
	3–4	64	40.8	92	36.4	1.15(0.74–1.80)	
	≥5	66	42	110	43.5	1	
Cigarette smoking							
	Yes	14	8.9	14	5.5	1	1
	No	143	91.1	239	94.5	0.59(0.27–1.29)	0.84(0.34–2.06)
At least one Chronic disease							
	Yes	36	22.9	63	24.9	1	
	No	121	77.1	190	75.1	1.14(0.69–1.78)	
Attitude							
	Positive	123	78.3	175	69.2	1	1
	Negative	34	21.7	78	30.8	0.60(0.39–0.98)	0.55(0.31–0.95)*
Knowledge							
	Good	102	65	120	47.4	1	1
	Poor	55	35	133	52.6	0.48(0.32–0.73)	0.59(0.36–0.95)*
Perceived Susceptibility							
	Low susceptibility	42	26.7	129	51	0.35(0.37–0.84)	0.33(0.20–0.54)***
	Highly susceptibility	115	73.3	124	49	1	1
Perceived Severity							
	Not severe	34	21.9	45	17.9	1.29(0.78–2.12)	
	Severe	121	78.1	207	82.1	1	
Perceived Self-efficacy							
	Low self-efficacy	42	26.8	67	26.5	1.04(0.64–1.59)	
	High self-efficacy	115	73.2	186	73.5	1	
Perceived Barriers							
	Barriers	20	12.7	49	19.4	0.68(0.34–1.06)	0.55(0.28–1.07)
	Not barriers	137	87.3	204	80.6	1	1
Perceived Benefits							
	Not Benefited	38	24.2	42	16.6	0.53(0.28–0.99)	0.88(0.42–1.87)
	Benefits	119	75.8	211	83.4	1	1

*p-value <0.05,

** p-value <0.001,

***p-value<0.0001;

# Government and private employed.

## Discussion

In this study an overall, 222(54.1%) [95% CI (49.3, 59.2%)] of the study participants have good knowledge towards COVID-19 and its prevention methods. This is similar to a study conducted at Northwest Syria (51%) [26]. However, it is lower than studies conducted across the globe: Southern Ethiopia 90% [21], Iran 90% [27], USA 71.7% [28], Nepal 79% [29], Uganda 84% [22], Italy 83.4% [30], Bangladesh 70% [23] and Paraguay’s 62% [31] demonstrated poor knowledge in related to the covid-19. The observed difference might be due to the socio-demographic and period of the study commencement in which most of the studies were conducted immediate to the pandemic. On the other hand it is higher than the magnitude reported from Thailand where, the majority, 73.4% of the study participants had poor knowledge of COVID-19 prevention and control [32]. Majority, 157 (38.5%) of respondents obtained information regarding COVID-19 from health personnel. One hundred sixty six (40.5%) of participants claimed that health personnel is trusted source of information. This is consistent with a study conducted at Kenya [33]. More than half, 246 (60%) of respondents mentioned that the main causes of COVID-19 is virus. More than three-in-five (62.6%) and more than three-in-fourth (78.5%) of the participants were responded that avoiding crowded place and frequent hand washing with soap and water/alcohol-based hand sanitizer as the main prevention methods of COVID-19.

Our study also identified factors affecting the knowledge level of participants about the coronavirus infection and its prevention mechanism. In our study the knowledge level of the participant was significantly higher among employed participants, who had positive attitude and those who had high perceived susceptibility of contracting the corona infection. This result is consistent with studies conducted in Southern Ethiopia [21] and Egypt [34] and China [25] in which participants with high socioeconomic status and hold optimistic attitudes were more knowledgeable about COVID-19.This might be associated with being employed has improved the prospect of sharing and seeking updated information about the COVID-19 and its prevention mechanisms. Moreover, a study conducted at Egypt also revealed result which supported our study [34].

The prevalence of Positive attitude, 298(72.7%) [95%CI:68.8%-76.6%] found in this study is comparable to a study done at Bangladeshi [23] and Paraguay’s [31] reported a desired attitude of the population towards COVID-19. However, the result is lower than studies conducted in Ethiopia [20,21]. This might be the difference in study participant’s characteristics, where the above studies majorities of the respondents were governmental employers. Pertaining to the factors affecting the attitude status of respondents, the odds of positive attitude was reduced among respondents who were in age group of 26–30 and 31–35 years as compared to respondents who were above 35 years of age. Likewise, respondents who had less perceived severity and low perceived self-efficacy were less likely to have positive attitude than their counter parts. This is congruent to a study report done at Addis Ababa, Ethiopia [20] and study conducted at Brazil on Health belief model for coronavirus infection risk determinants [35]. The possible explanation might be due to the reason that having perception of greater severity may lead the community to seek health services earlier.

In this study, 38.3% **[95% CI: 33.5%, 43.1%)]** of the study participants have good social distancing practices for the prevention of COVID-19. This result is supported by a study done in Thailand [32], Bangladesh [23], Kenya [33] and United Kingdom [24]. However, the result is incomparable or lower than studies conducted at Uganda [22]. The observed difference might be due to the difference in timing of the study, the distribution of the outbreak across the nation cities or towns and socio-demographic characteristics of the participants. Moreover, this study was conducted at the time where different governmental sanctions were lifted off; particularly state of emergency was completely removed.

Concerning the social distancing; less than half, 47.8% of respondents maintained 2 meters distance between themselves & other people. This is higher than studies conducted in Northwest Syria, 17% [26] and Nigeria, 20.4% [36]. The possible explanation could due the difference in socio-demographic characteristics and study period. However, our study result is lower than a study conducted at Addis Ababa, Ethiopia where, 89.5% of respondents practiced physical distancing [20], Italy 85.6% [37], Southern Ethiopia 65% [21]. The possible explanation for this lower practice could be due to the participant’s behavior of adopting the newly introduced rules and regulations. Furthermore, the participant’s natures in the aforementioned studies were urban compared to our study participants. So, this might contributes for the observed lower social distancing practices in our study.

On the other hand, the study result revealed that nearly three in-five (62.4%) of respondents never avoided non-essential use of public transport. This is comparable to study done in Iran (61.8%) [27], South Korea [38] and Addis Ababa, Ethiopia [20]. In our study more than five in-six, (84.9%) of respondents maintained non-contact greeting. This is comparable to a Addis Ababa, Ethiopia [20]. The possible explanation for this high practice could be the participant’s knowledge on the mode of transmission of the disease.

The odds of good social distancing practice was 45% reduced among household members who have negative attitude towards social distancing practices compared to their counter parts. This finding is supported by other studies conducted in Brazil [39], Hong Kong, China [40] and Bangladesh [23]. This is because an individuals who have positive attitude may have better social distancing practice for COVID-19 preventive measures than the individual who have negative attitude. Respondents who have poor knowledge about social distancing practices have 41% reduced social distancing practices as compared to respondents who have good knowledge about the social distancing practices. This finding is supported by the studies carried out in Hubei, China [25], and Pakistan [41]. This is due to the fact that, respondents who have knowledge on COVID-19 cause, mode of transmission, symptoms and prevention methods would be more likely to practice social distancing.

Odds of good social distancing practices was 67% reduced among individuals who have low perceived susceptibility of contracting COVID-19 as compared to individuals who have high perceived susceptibility of contracting COVID-19. This finding is corroborated by studies conducted in Hong Kong, China [40], South Korea [38] and worldwide survey [42]. Likewise a large survey conducted in 48 countries also reported similar result [42]. This indicates that the perceived level of personal susceptibility has created fear when seeing hard-hitting emotional messaging. As a result individuals became aware and adhere to social distancing practices to reduce perceived threat. Moreover, current evidence showed that respondents with high behavioral responses found to be practicing social distancing.[38].

Those respondents who were employed were 6 times more likely to adhere to social distancing practice as compared to those who were unemployed. The Similar findings were reported by studies conducted in Addis Ababa, Ethiopia [20], Southern Ethiopia [21], Bangladesh [23], Brazil [43] and Uganda [22]. This could be due to the awareness and daily exposure of information, enforcement of social distancing practices within work environment. Likewise, at the time of the incidence of the pandemic the majorities of organization were permitted their staffs to work at their home and also reduced the number of staff working on daily basis. This would also by itself minimize the use of public transportation and unnecessary gatherings. More importantly, individuals who were more educated or employed would have a greater tendency to engage in protective behaviors during pandemics.

The age of respondents was also positively associated with social distancing practices. The odd of good social distancing practices was higher among respondents who were in the age group of 26–30 and 31–35 years as compared to respondents who are above 35 years of age. This is consistent with a study conducted at Malaysia reported that those older age were more likely to attend daily religious ceremonies [44]. This similarity might be due to the socio-demographic characteristics of the participants, where in both study area the majority of communities are religious. In contrary, a study conducted at United Kingdom revealed that aged 70 and above had good social distancing practice measures [24]. The observed difference might be difference in demographic size and composition among the study areas.

### Limitations and strength of the study

Among the strengths of our study; first this is the first study conducted at the study area. Second, the study included all the kebeles found in the study town, Third; it is a community based study which enables us to generalize our findings for our source population. Despite its strength, the limitations of our study are: the timing of the study conducted where the attention of the COVID-19 had been decreased. The introduction of social desirability biases particularly on social distancing related variables, and lastly, the cross-sectional nature of the study design does not establish the cause and effect relationship.

## Conclusion

This study results showed that the smaller proportion of the study participants had demonstrated good knowledge, and good social distancing practice. Individuals should abide and implement the information released from regional health bureau and FMOH. Moreover, Bule Hora Town Health Office and West Guji Zone Health Department should give emphasis on providing continues awareness creation so as to lift the knowledge of the community, particularly on the mechanisms of covid-19 prevention techniques due stress on social distancing practices. Although, the results of this study can be used as baseline information for the local, regional and national governments and other stakeholders engaged in the prevention and control of COVID-19, further study should be conducted to get more representative data for the policy makers and triangulate with qualitative to explore other different possible determinant factors.

## Supporting information

S1 File English version questionnaire. (PDF)

S2 File Raw SPSS dataset. (ZIP)
