# Supplementary material for: Social distancing practice and associated factors in response to COVID-19 pandemic at West Guji Zone, Southern Ethiopia, 2021: A community based cross-sectional study
Source: PLoS One. 2021 Dec 20;16(12):e0261065. doi: 10.1371/journal.pone.0261065 (PMC8687536; doi:10.1371/journal.pone.0261065)
Supplement: S1 File — (PDF) [file pone.0261065.s001.pdf]

Participant information sheet and informed consent form

Good morning/afternoon dear participant! My name is _________________. I am working as a data collector for the study being conducted in this Health facility on the magnitude of social distancing practice and associated factors among residents of Bule Hora Town by instructors of Bule Hora University, College of Health and Medical Science. I kindly request you to lend me your attention to explain about the study and being you selected as the study participant.

The study title: Based Social Distancing Practice and Associated Factors in Response to COVID-19 Pandemic at Bule Hora Town, Southern Ethiopia, 2020

Purpose of the study: to identify the compliance of community to non-pharmaceutical intervention during COVID 19 pandemic in Bule Hora Town

Procedure and duration: majority of questions will be responded orally; Data collection stays for one month.

Risk and benefits: the questions will take your time and there may be a little discomfort. There is no direct benefit from study but in directly you will get benefit when our country's development improved.

Confidentiality: The information collected about you will be accessed only by the researcher and research assistant. For this matter we will use number code instead of writing your name.

Rights: participation is based on only your decision; you can either to participate or not. You can also withdrawal at any time if you feel discomfort and your refusal to participate do not cause any harm.

Contact address of PI:

Name of PI: Mr. **Anteneh Fikrie** Phone number: 0922465129

Co-investigator: **Elias Amaje** Phone number: 0916648950

Declaration of informed voluntary consent:

I have read and understood all the process, objective, purpose, advantage, and disadvantage of the study entitled “Social Distancing Practice and Associated Factors in Response to Covid-19 Pandemic at Bule Hora Town, Southern Ethiopia”. I have been told that the study imposes no risk, participation is completely voluntary based and I will free to drop it any time if I fill discomfort. I have told and understood no compensation would be provided to me for my participation and my answers to the questions will not be given to anyone. So, I agreed to give my consent to participate in the study voluntarily.

Interviewer: shall I continue? 1. Yes 2. No, Stop and thank the respondent.

Witness: Signature____________ Date _____________ Data collector

English Version Questionnaire for participant interview

**Date of interview--------------------**	**Code -----------------**
**Name of interviewer ________________________**<br>**Sign________**	**Kebele ____________**

Section I: Socio demographic and economic characteristics

No.	Question	Response	Skip to
100	Age	----------Years	
101	Sex	1. Male 2. Female	
102	Education status	1. Unable to read and write<br>2. Write and read only<br>3. Primary Completed<br>4. Secondary completed<br>5. College and above	
103	Marital Status	1. Married<br>2. Single<br>3. Divorced<br>4. Widowed	
104	Religion	1. Muslim<br>2. Orthodox<br>3. Protestant<br>4. Waqefeta<br>5. Others (specify)___________	
105	Occupational status	1. Government employed<br>2. Merchant<br>3. Farmer<br>4. NGO employed<br>5. Private<br>6. Housewife<br>7. Others (Specify)______	
106	What is the average monthly income of the family?	________________ in birrs	
107	Housing tenure (ownership)	1. Private<br>2. Rented<br>3. Other (specify) _________	
108	No of family size	__________	

Section II. Chronic medical history of the participants			
No.	Question	Response	Skip to
200	Have you been diagnosed with Diabetes Mellitus?	1. Yes 2. No 3. I don't know	
201	Have you been diagnosed with Hypertension?	1. Yes 2. No 3. I don't know	
202	Have you been diagnosed with cardiac disease?	1. Yes 2. No 3. I don't know	
203	Have you been diagnosed with Asthma?	1. Yes 2. No 3. I don't know	
204	Have you been diagnosed with Cancer?	1. Yes 2. No 3. I don't know	
205	Have you been diagnosed with HIV/AIDS?	1. Yes 2. No 3. I don't know	
206	Do you have a chemotherapy (treatment of cancer)	1. Yes 2. No 3. I don't know	

Section III: Individual risk perceptions about the current COVID-19 Pandemic						
1	**Perceived susceptibility**	**Responses**				
		**S. disagree**	**Disagree**	**Neutral**	**Agree**	**S. agree**
	1. Less chance to transmit infection to family members from sick person?					
	2. No chance to get infection for healthy person					
	3. Little chance to get infection for young					
	4. High chance to get infection for abroad/ together with foreigner					
	5. Easily get disease in crowded place					
	6. Healthy life style will reduce the chance of infection					
2	**Perceived severity**	**S. disagree**	**Disagree**	**Neutral**	**Agree**	**S. agree**
	1. COVID-19 will be more serious among elderly and people with comorbidities?					
	**2.** If I were infected with covid-19, I will suffer severe symptoms					
	3. If I were infected with covid-19, I could not survive					
	4. I can suffer from covid-19 without signs and symptoms					
	5. Covid-19 will be treated if I were infected					
	6. If I were infected with Covid-19, i will recover spontaneously					
3	**Perceived self-efficacy**	**No**	**Low confident**	**Neutral**	**Much**	**High confident**
	1. I can get access to the reliable health information on covid-19					
	2. I will eat healthy diet to prevent covid-19					
	3. To prevent covid-19, I will wash my hands					
	4. I can prevent COVID-19					

	5. To prevent covid-19, I will avoid visiting crowded places					
	6. To prevent covid-19, I will use face mask whenever I go to crowded place					
4	**<u>Perceived barriers</u>**	**S. disagree**	**Disagree**	**Neutral**	**Agree**	**S. agree**
	1. It is hard **<u>refraining</u>** social gatherings in my home?					
	2. It is hard to stay home too much?					
	3. It is difficult using face mask daily?					
	4. Can't afford to buy soap/alcohol containing hand sanitizer					
5	**<u>Perceived benefits</u>**	**S. disagree**	**Disagree**	**Neutral**	**Agree**	**S. agree**
	1. When I am doing something protective measures of covid-19, I am caring for myself and my families					
	2. When I keep social distancing, I am setting a good example for others					
	3. When I wear face mask at crowded area, I am decreasing my chances of contracting covid-19?					
	4. Staying home will reduce my chances of contracting covid-19?					

**Section IV. Questions assessing participants' ATTITUDE about Social Distancing**						
S.No	Questions	**Responses**				
		**S. disagree**	**Disagree**	**Neutral**	**Agree**	**S. agree**
1	Do you like to stay at home for certain period (14 days) to prevent covid-19 spread if government will order so?					
2	Do you agree that being black race is protective towards CIVID-19?					
3	Do you think that social distancing (e.g. stay 2 m apart, avoiding crowds, etc.) can prevent covid-19 spread?					
4	Do you agree that we should cancel business/recreational trips at this time?					
5	Do you believe that working from home can help to control covid-19?					
6	Do you agree that government has taken sufficient preventive measures to prevent the spread of covid-19?					
7	Wearing a well-fitting face mask is effective in preventing COVID-19?					
8	Hand washing with water and soap/ alcohol based hand sanitizer can prevent you from getting covid-19					
9	When someone has signs and symptoms of COVID-19, I can confidently keep my physical distance from him/her?					
10	Do you think that, Ethiopia is in a good position to **contain** COVID-19?					

**Section V Participants Knowledge towards COVID-19**			
S.No	Questions	Responses	Skip
1	Have you heard about corona virus?	1. Yes 2. No	If No, go to Q4
2	If Yes to the above question, what was your source of information on COVID-19? Tick all that apply.	1) Healthcare personnel<br>2) Social media (Facebook/websites)<br>3) FMOH sources<br>4) Mass media (radio/TV/newspaper)<br>5) Friends/family members/relatives<br>6) Others, specify____________________	
3	Which of the followings was your most trusted source of information?	1) Healthcare personnel<br>2) Social media (Facebook/websites)<br>3) FMOH sources<br>4) Mass media (radio/TV/newspaper)<br>5) Friends/family members/relatives<br>6) Other, specify____________________	
4	What is the cause of COVID-19?	1) Virus 3. Bacteria<br>2) Fungus 4. Protozoa<br>5). I don't know	
5	Can COVID-19 transmit human-to-human?	1. Yes 2. No	
6	What are the modes of transmission of COVID-19? **More than one answer is possible**	1) Airborne (other people coughing, etc.)<br>2) Drinking/washing in infected water<br>3) Physical contact with contaminated object<br>4) Physical contact with infected people<br>5) Eating raw meat/animal products<br>6) Other (specify………	
7	What are the simple everyday preventive actions to COVID-19? (more than one answers- **Do not probe)**	1) Avoid close contact with people who are sick<br>2) Frequent hand washing with soap and water/ alcohol-based hand sanitizer<br>3) Avoid touching your eye, nose, mouth with unwashed hands<br>4) Avoid shaking hands<br>5) Avoid crowded place	

		6) Disinfecting/ cleaning objects and surfaces<br>7) Stay at home/work at home<br>8) Practicing good respiratory hygiene<br>9) Others, specify__________________	
8	What are the main clinical symptoms of COVID-19? **(Tick all that apply)**	1) Fever<br>2) Dry Cough<br>3) Breathing Difficulty<br>4) Fatigue<br>5) Sneezing<br>6) Headache	
9	Is there effective vaccine for COVID-19?	1) Yes 2. No 3. I don't know	
10	Is there any definitive treatment of COVID-19 currently?	1. Yes 2. No 3. I don't know	
11	Do you know the high-risk population for contracting COVID-19? **(More than one answers. Do not probe)?**	1) Everyone<br>2) Children<br>3) Elderly<br>4) Pregnant /lactating women<br>5) People with chronic disease (like; DM, HIV, HTN, Asthma, Cancer)<br>6) Smoker<br>7) health workers	
12	Isolation and treatment of people who are infected with the COVID-19 virus are effective ways to reduce the spread of the virus.	1) Yes 2. No 3. I don't know	
13	People who have contact with someone infected with the COVID-19 virus should be immediately quarantined in a proper place for at least 14 days of period?	1) Yes 2. No 3. I don't know	

Section VI: Participants Social distancing practice related questions				
S.No	Questions	Responses		
		Always	Occasional	Never
1	How often do you avoid contact with someone who is displaying symptoms of coronavirus?			
2	Do you avoid non-essential use of public transport when possible?			
3	How often do you Work at home?			
4	Do you avoid large and small gatherings in public spaces (pubs, restaurants, leisure centers)			
5	Do you avoid gatherings with friends and family			
6	Maintaining non-contact greetings			
7	Do you Maintaining a 2 meters distance between yourself & other people			
8	Do you stay home when you ill?			
