# Supplementary material for: Social distancing practice and associated factors in response to COVID-19 pandemic at West Guji Zone, Southern Ethiopia, 2021: A community based cross-sectional study
Source: PLoS One. 2021 Dec 20;16(12):e0261065. doi: 10.1371/journal.pone.0261065 (PMC8687536; doi:10.1371/journal.pone.0261065)
Supplement: Attachment — Submitted filename: comments.docx [file pone.0261065.s003.docx]

1- **Study setting (methodology section)**

Spelling mistake in line 3

2- **Explain the sampling technique in methodology section (no sampling technique)**
3- **What is the aim of calculating sample size for each objective separately?**
4- **What is meant by kebele?**
5- **Why you apply pilot testing on 5% of the sample?**
6- **Why you consider 50% as a cut off point for dividing the people into good or poor in knowledge, attitude, Social distancing practice, ------etc?**

As these are means, you can consider those who have above the mean as good and below the mean as poor

7- **More explanation of the data collection tool is needed regarding knowledge, attitude, Social distancing practice, ------etc?**
8- **Rewriting of the references is needed**
9- **What is meant by Merchant in the occupational status (should be mentioned)?**
10- **What is currency of the monthly income?**
11- **What is the value of asking about housing tenure, TV and Radio in sociodemographic characteristics?**
12- **In this study, 38.3% [95% CI:33.5%, 43.1%)] of the study participants have good social distancing practices for the prevention of COVID-19.**

Where does this finding present in your results?

13- **The title of the manuscript should be broadened because social distance practice and associated factors are present in table 10, 11 only.**
