# Supplementary material for: Social distancing practice and associated factors in response to COVID-19 pandemic at West Guji Zone, Southern Ethiopia, 2021: A community based cross-sectional study
Source: PLoS One. 2021 Dec 20;16(12):e0261065. doi: 10.1371/journal.pone.0261065 (PMC8687536; doi:10.1371/journal.pone.0261065)
Supplement: Attachment — Submitted filename: 2. Point-by-point response to reviewers file.docx [file pone.0261065.s004.docx]

9 June, 2021

Dear respected reviewers,

***Ref: A point-by-point response to the comments***

**Dear reviewer 1**, First of all I would like to thank you very much for your appreciation and forwarding constructive and insightful comments which we believe that it would improve the quality of our manuscript. Thus, according to your comments, suggestions and questions the following amendments has been taken.

**Amendments done on the minor comments raised by reviewer-1**

**1- Study setting (methodology section) Spelling mistake in line 3**

**Answer:** I have corrected the mistake

**2- Explain the sampling technique in methodology section (no sampling technique)**

**Answer:** I have included the sampling technique under “***Sample size determination and sampling procedure” section paragraph two line 7 and 8.***

3. **What is the aim of calculating sample size for each objective separately?**

**Answer:** Sample size calculation for the first objective helps us to estimate the magnitude of social distancing and calculated using single population proportion formula. On the other hand, sample size calculation for the second objective has been determined by using a double population proportion for considering the possible factors associated with social distancing. In fact, the largest sample size was obtained from the second objective. As the sample size increases, the confidence in our estimate increases

**4- What is meant by kebele?**

**Answer:** According to Ethiopia Kebels are the lowest administrative units. I have included this sentence in to the mother document to the methods section and highlighted.

**5- Why you apply pilot testing on 5% of the sample?**

**Answer:** Pretesting survey questionnaire is one of the mechanisms of maintaining the quality of the data. It will reduce the measurement errors, reduces respondents burden, helps us to know whether or not participants are interpreting questions appropriately, and ensure us the order of questions are not influencing the way a participants answers. Therefore, in case if there is a problem stated above could be easily identified and appropriate remedial would be taken. Fortunately, nothing has obtained after pilot result.

6- Why you consider 50% as a cut off point for dividing the people into good or poor in knowledge, attitude, Social distancing practice, ------etc? As these are means, you can consider those who have above the mean as good and below the mean as poor.

**Answer:** Mean is the best measure of the central tendency to be used when you the distribution of the data is symmetrical.

**7- More explanation of the data collection tool is needed regarding knowledge, attitude, Social distancing practice, ------etc?**

**Answer:** I have explained.

**8- Rewriting of the references is needed**

**Answer:** I have corrected

**9- What is meant by Merchant in the occupational status (should be mentioned)?**

**Answer:** Merchant means trader. I have included in the table.

**10- What is currency of the monthly income?**

**Answer:** The currency of the monthly income of the participants is Ethiopian birr (1 birr=0.0229$). I have corrected at the table also**.**

**11- What is the value of asking about housing tenure, TV and Radio in socio-demographic characteristics?**

**Answer:** TV and Radio are the commonest source of information for Ethiopian communities. As a result, I have asked participants about TV and Radio to know whether or not the source of information about COVID-19 and its prevention mechanisms is from mass media. Likewise, asking about housing tenure of the participants was due to the fact that individuals who have private owned house has higher likelihood of staying at home as compared to individuals who lived in rental house.

**12- In this study, 38.3% [95% CI:33.5%, 43.1%)] of the study participants have good social distancing practices for the prevention of COVID-19. Where does this finding present in your results?**

**Answer:** **The above finding is present at page number 11, line number 1 under the section entitled “*****Social distancing Practice of Study Participant for COVID-19 Prevention”***
