# Supplementary material for: Social distancing practice and associated factors in response to COVID-19 pandemic at West Guji Zone, Southern Ethiopia, 2021: A community based cross-sectional study
Source: PLoS One. 2021 Dec 20;16(12):e0261065. doi: 10.1371/journal.pone.0261065 (PMC8687536; doi:10.1371/journal.pone.0261065)
Supplement: Attachment — Submitted filename: Step by step responses to reviewers Second Revision.docx [file pone.0261065.s005.docx]

November 12, 2021

**Manuscript ID**: PONE-D-21-13624

**Manuscript title:** Social Distancing Practice and Associated Factors in Response to COVID-19 Pandemic at West Guji Zone, Southern Ethiopia, 2020 A Community Based Cross-sectional study

Dear respected reviewers,

***Ref: A point-by-point response to the comments***

**Dear reviewer** First of all I would like to thank you very much for your appreciation and forwarding constructive and insightful comments which we believe that it would improve the quality of our manuscript. Thus, please find in the table below the amendments and responses based on your comments, suggestions and questions. We hopefully believe that the current version of our manuscript has met the concerns of the reviewers and your editorial team.

| Number | Questions/comments from reviewer | Answer from author | Lines |
| --- | --- | --- | --- |
| 1 | **Introduction**   - Can you please show us the importance clearly of social distance (SD) in the covid-19 limitation of transmission without using face mask and in which percentage the SD itself can limit the disease propagation in the community? | I thank you really for this comment. I have incorporated the sentence accordingly. | **99-106** |
| **2** | - Add literature in the introduction about research done in Africa bout KAP showing the implication of social distance on limitation of COVID-19 transmission | Thank you your insightful comment. I have added some literatures according to your comments. | 107-113 |
| **3** | - You conclude the introduction by showing the purpose of the study but in which situation was this study carried out since the some countries in the African continent have underwent series of lockdown as a major means to limit the disease spread, can please the authors provide the time in which the country as a justification of this study? | Thank you for the question. The revised draft of the manuscript has been modified. | **114-121** |
| **4** | **Methods**   - Is it possible to talk about the place where the study was carried out in term of activities and some more detail of the region in the methodology? | Thanks very much for your concern. I have reduce the details of the region | **125-128** |
| **5** | - Please try to deeply describe the sampling technique in this study, which technique was used? | We have employed simple random sampling technique and tried to describe in the manuscript document. | **160-166** |
| **6** | - How and why the sample size was not got in 100% (410 instead of 447??); | I have described this in the result section. The study response rate was 410 (91.7%), this mean that the remaining 37(8.3%) were not voluntarily to be participated in the study. | **259-260** |
| **7** | - Describe properly and with detail the population included in the study and the place of data collection according to geographical distribution per km/population | I have described this in the methods section under Study population, sample size determination and procedure sub-section | **143-146** |
| **8** | - You mentioned that the questionnaire was made in English and translate in local language, what was the power of validating this questionnaire after translation?? and then after collecting the data was the questionnaire translated back in English or not? if yes, then please mention it in the text. | The questionnaire was translated back in to English. I have mentioned this statement in the methods section. | **174** |
| **9** | - **The result is not well presented and the knowledge is not about social Distance** but about COVID-19, can the authors please revise the result according to the main objective of the study and discuss accordingly (demographics, KAP, Factors and bivariate and multivariate analyses; **noted** that bivariate and multivariate in the same table per group vis via KAP and factors). | Dear respected reviewer, thank you very much for your insightful comments you raised here. I have presented the results according to your comments. | **304-372** |
| **10** | - **Discussion should be presented accordingly too.** | Thank you again for your constructive comments. I have presented the discussion based on your comments. | **376-480** |
| **11** | - Please, provide limitations and strength of this study at the end of the discussion | Thank you. I have incorporated the limitations and strength of the study. | 481-488 |
| **12** | - Please do not repeat the result in the conclusion but provide the take away message from this study and suggestions to local people, government and future researchers | Thank you for your politeness. I have wrote the conclusion without repeating the result. | 490-500 |
