# Supplementary material for: Social distancing practice and associated factors in response to COVID-19 pandemic at West Guji Zone, Southern Ethiopia, 2021: A community based cross-sectional study
Source: PLoS One. 2021 Dec 20;16(12):e0261065. doi: 10.1371/journal.pone.0261065 (PMC8687536; doi:10.1371/journal.pone.0261065)
Supplement: Attachment — Submitted filename: Step by step responses to reviewer-2 Third revision.docx [file pone.0261065.s006.docx]

November 15, 2021

**Manuscript ID**: PONE-D-21-13624R2

**Manuscript title:** Social Distancing Practice and Associated Factors in Response to COVID-19 Pandemic at West Guji Zone, Southern Ethiopia, 2020 A Community Based Cross-sectional study

**Dear respected reviewer-2,**

It is my pleasure to bestow my gratitude for your genuine appreciation on our second revised manuscript document. Moreover, the comments you gave us yet would also improve the quality of our manuscript. Thus, please find in the table below the amendments and responses based on your insightful and constructive comments, and suggestions.

| Number | Questions/comments from reviewer | Answer from author | Lines |
| --- | --- | --- | --- |
| 1 | **In the introduction,**   - Please update the figures of COVID-19 cases (Lines 69-70) | Really, I would like to forward a big salute for this comment. I have updated the figures according to the following. As of 1 December, more than 254 million cases and 5.1 million deaths have been reported globally until 15 November 2021 | 69-70 |
| 2 | **Methodology:**   - Please arrange this part as per PlosOne guideline and also remove the ethical part (lines 134-139) in the study period and population parts but put it in a separate part named "Ethical approval" | Thank you again for your comment. I have removed the ethical consideration part and put as a separate subheadings below to the data processing and analysis section | 246-252 |
|  | - Also the remaining part meaning lines 139-143 in data collection procedures and quality part | Here also, I have moved and included the remaining sentences under the data collection procedures and quality section. | 183-186 |
| 3 | Please write the word COVID-19 in capital throughout the text | I have appreciated your wonderful, deep and scientific comments. I do have changed all the sentence letter to capital letter for the word COVID-19 throughout our manuscript |  |
